# A Common R219K Variant of ATP-Binding Cassette Transporter A1 Gene Alters Atherometabolic Traits in Pregnant Women With Gestational Diabetes Mellitus

**DOI:** 10.3389/fendo.2021.782453

**Published:** 2021-12-17

**Authors:** Fangmei Tang, Linbo Guan, Xinghui Liu, Ping Fan, Mi Zhou, Yujie Wu, Rui Liu, Yu Liu, Sixu Liu, Dehua Li, Huai Bai

**Affiliations:** ^1^ Laboratory of Genetic Disease and Perinatal Medicine and Key Laboratory of Birth Defects and Related Diseases of Women and Children of the Ministry of Education, West China Second University Hospital, Sichuan University, Chengdu, China; ^2^ West China School of Nursing, Sichuan University, Chengdu, China; ^3^ Department of Obstetrics and Gynecology, West China Second University Hospital, Sichuan University, Chengdu, China; ^4^ Division of Peptides Related With Human Disease, West China Hospital, Sichuan University, Chengdu, China; ^5^ Department of Biochemistry and Molecular Biology, West China School of Preclinical and Forensic Medicine, Sichuan University, Chengdu, China

**Keywords:** *ABCA1* gene, single nucleotide polymorphism (SNP), gestational diabetes mellitus, obesity, atherometabolic traits

## Abstract

**Background:**

ATP-binding cassette transporter A1 (ABCA1) has important roles in high-density lipoprotein (HDL) metabolism and reverse cholesterol transport, and is implicated in lipid-related disorders. Genetic variants are involved in the pathogenesis of gestational diabetes mellitus (GDM). The objective of this study was to investigate the association of rs2230806 (R219K), a single nucleotide polymorphism (SNP) in the lipid-related gene, with the risk of GDM and related traits.

**Methods:**

The SNP, rs2230806, was genotyped, and clinical and metabolic parameters were determined in 660 GDM patients and 1,097 control subjects. Genetic associations with related traits were also analyzed.

**Results:**

The genotype distributions were similar in GDM patients and normal controls. However, significant differences in the variables examined in the study subjects were noted across the three genotypes. The genotype at the rs2230806 polymorphism was significantly associated with HDL-cholesterol (HDL-C) levels and atherogenic index (AI) values in GDM patients and total cholesterol (TC) and low-density lipoprotein-cholesterol (LDL-C) levels in control subjects. Subgroup analysis showed that the polymorphism was associated with diastolic blood pressure, in addition to HDL-C levels and AI, in overweight/obese GDM patients, while it was associated with TC levels, AI, pre-pregnancy body mass index (BMI), and BMI at delivery in non-obese GDM patients. In addition, this polymorphism was associated with TC, LDL-C, and apoB levels in overweight/obese control subjects.

**Conclusions:**

The rs2230806 polymorphism in the *ABCA1* gene was associated with variations in atherometabolic traits in GDM patients, with characteristics of BMI dependency, but not with GDM. Our findings highlight a link between related phenotypes in women with GDM and genetic factors.

## Introduction

Gestational diabetes mellitus (GDM) is the most common metabolic disorder that occurs during pregnancy. It is defined as glucose intolerance beginning in the second or third trimester of pregnancy (1). The prevalence of GDM varies between 1% and 28% globally (2), and is especially prevalent in developed countries (3). The reported prevalence of GDM in pregnant Chinese women is also high, with a rate of 14.8% (4). The increased incidence of risk factors, such as obesity, poor dietary habits, physical inactivity, and older maternal age, has led to an increased occurrence of the disease globally in recent years (4, 5). GDM can be dangerous to mothers and infants in the perinatal period (5), and it is associated with an increased risk of the development of metabolic-related disorders, such as type 2 diabetes mellitus, metabolic syndrome, and cardiovascular diseases in the years following pregnancy (5, 6). The etiology of GDM may involve genetic factors (7), lipid abnormalities (8), oxidative stress (9), and inflammatory reactions (10).

The *ABCA1* gene is located on the human chromosome 9q31 and it encodes an integral cell membrane protein that facilitates the efflux of cholesterol and phospholipids. ABCA1 is highly expressed in various human tissues, including the placenta, uterus, liver, adrenal glands, small intestine, lungs, and heart (11), and it is believed to be functionally relevant in these tissues. In addition, activated pancreatic beta cell ABCA1 would stimulate insulin production. One study demonstrated that GDM affects different key players of cholesterol transfer including ABCA1, in placenta depending on the maternal BMI (12). Another study reported that up-regulation of ABCA1 and ABCG1 mRNA and protein levels were observed in GDM versus control human fetoplacental endothelial cells (13). In addition, recent study evaluated effects of GDM and obesity on the intrauterine metabolic milieu in the fetus, showing exposure to maternal obesity *in utero* leads to sex-specific changes in miRNA and expression of several target genes, including ABCA1 in human fetal liver (14). Furthermore, one study showed that serum ABCA1 could be detected in women with normal pregnancy, and the patients with preeclampsia had significantly lower serum ABCA1 levels than those in the control subjects (15). A substantial number of studies have shown a new role for ABCA1 in the liver and a metabolic relationship between increased TG levels and decreased high-density lipoprotein (HDL) levels (16, 17). Moreover, recent studies have investigated the role of ABCA1 in other features of metabolic syndrome and demonstrated its relationship with body weight, decreased insulin secretion and sensitivity, and increased blood glucose levels (18–20). Changes in these metabolic components are also frequently encountered in pregnant women, especially in pregnant women with GDM. In addition, ABCA1 has been reported to be expressed at high levels in various compartments of the placenta, including the villous syncytiotrophoblasts and fetal endothelial cells (21). ABCA1 is also linked to the feto-maternal transport of cholesterol, indicating its crucial role in normal fetal development in humans. Several studies of the human placenta have shown that ABCA1 exerts important beneficial effects against cardiovascular and metabolic diseases, and thus, is still of major interest as a therapeutic target for the prevention of such disorders. Approaches to targeting ABCA1 include the upregulation of ABCA1 expression by LXR activators (22) and recently discovered specific microRNAs (23).

Since ABCA1 has demonstrated roles in various GDM-related traits, such as lipid levels, obesity-related phenotypes, and insulin secretion and sensitivity, it may be a strong candidate gene for predisposition to GDM. The *ABCA1* gene is highly polymorphic. One of the most common single nucleotide polymorphisms (SNPs) is the rs2230806 SNP (also known as R219K or G1051A) in exon 7. This SNP results in the substitution of an arginine amino acid (G allele) with a lysine (A allele) amino acid at position 219 of the polypeptide. This amino acid substitution is functionally relevant to the role of ABCA1 in lipid metabolism. For example, the A allele of the SNP is associated with higher levels of HDL-cholesterol (HDL-C) in various populations, such as Turkish (24), European (25), Japanese (26), and Chinese (27) populations. In addition, the A allele of the SNP is reported to be significantly associated with lower triglyceride (TG) (24, 28), total cholesterol (TC) (29–31), and low-density lipoprotein cholesterol (LDL-C) (28, 32, 33) levels. Clinically, the allelic variant of this SNP has been found to be associated with a reduced incidence of coronary artery disease (CAD) events (25, 34), premature CAD in patients with familial hypercholesterolemia (35), and type 2 diabetes mellitus (36). We speculated that there may be a relationship between this *ABCA1* SNP and the risk of GDM and/or related traits or phenotypes.

We tested the hypothesis that ABCA1 rs2230806 SNP genotype is associated with the risk of GDM and/or atherometabolic traits in GDM patients. To date, there is little information on the possible relationship between *ABCA1* polymorphisms and GDM. In this study, we evaluated the distribution of the *ABCA1* rs2230806 SNP genotypes in a relatively small sample size of pregnant women with (n = 660) or without (n = 1,097) GDM from a well-characterized Chinese population. We also investigated whether this SNP was associated with the risk of GDM and related traits in our study subjects.

## Materials and Methods

### Study Subjects

Blood samples were taken from 1,757 Chinese women from 2013–2020, including 660 patients (average age, 35.57 ± 4.03) with GDM and 1,097 control subjects (average age, 34.54 ± 4.99). All study subjects were of Chinese Han ethnicity. This study was conducted in accordance with the principles of the Declaration of Helsinki. Written informed consent was obtained from all participants. The study was approved by the Institutional Review Board of West China Second University Hospital, Sichuan University (No. 2017–033).

All patients with GDM were recruited from the Obstetrics Department of West China Second University Hospital at Sichuan University in Chengdu. Each patient with GDM met the diagnostic criteria recommended by the International Association of Diabetes and Pregnancy Study Groups (24-28 weeks of gestation, fasting blood glucose concentration ≥ 5.1 mmol/L by oral 75 g glucose tolerance test, blood glucose concentration ≥ 10.0 mmol/L 1 h after and ≥ 8.5 mmol/L 2 h after glucose consumption) (37). Control subjects with uncomplicated pregnancies were recruited from the same department of the hospital during the same period. Patients were included if they underwent elective cesarean section or planned vaginal delivery at term, and had a singleton pregnancy. None of the study subjects had clinically evident chronic or acute diseases, such as infections, tumors, thyroid dysfunction, or cardiovascular disease.

Body weight in light clothing and barefoot height were measured, and body mass index (BMI, kg/m^2^) was determined. Normal weight and overweight/obesity were defined as BMI < 25 and ≥ 25 kg/m^2^, respectively. Sitting blood pressure was measured on the right arm after a 10-min rest with a semi-automatic device (Omron HBP-1300). The infant’s length and weight were measured after birth. Blood samples of the study subjects were taken in a stage of late pregnancy after an overnight fast. Plasma aliquots were stored at -80°C.

### DNA Extraction and Genotyping

Genomic DNA was isolated from 500 μL of peripheral blood according to the method described by Erlich (38). PCR was performed in a final volume of 25 μL containing 12.5 μL of 2× Taq PCR Master Mix, 10.5 μL of ddH2O, and 0.5 mM each upper- and lower-strand primer. We used 1.0 μL of DNA sample per PCR. The investigated DNA sequences were amplified using the forward primer, 5′-GTA TTT TTG CAA GGC TAC CAG TTA CAT TTG ACA A-3′ and the reverse primer, 5′-GAT TGG CTT CAG GAT GTC CAT GTT GGA A-3′, which produced a 174 bp product. Amplifications were performed using a MyCycler™ thermal cycler system (Bio-Rad, Hercules, CA, USA) under the following conditions: initial denaturation at 94°C for 4 min, followed by 32 amplification cycles of denaturation at 94°C for 50s, primer binding (annealing) at 58°C for 50s, and chain elongation at 72°C for 1 min. The PCR program ended with a 10-min chain elongation step at 72°C. PCR products were digested with 10 U/μL Styl-HF (New England Biolabs, Ipswich, MA, USA), analyzed by electrophoresis on a 2.5% agarose gel, and visualized by staining with Genecolour fluorescent dye. Enzyme digestion resulted in 109- and 65-bp fragments for the GG genotype; a non-digested 174-bp fragment for the AA genotype; and 174-,109-, and 65-bp fragments for the GA genotype. To document the results, gel images were captured under ultraviolet light.

### Analysis of Metabolic Parameters

Plasma insulin levels were measured using chemiluminescence assays (IMMULITE 2000; Diagnostic Products Corporation, Los Angeles, CA, USA). The concentrations of TC, HDL-C, LDL-C, and TGs were measured in plasma samples using an enzyme assay (Boehringer, Mannheim, Germany), and serum apoA1 and apoB levels were determined by polyethylene glycol-enhanced immunoturbidimetric assay (Siemens Healthcare Diagnostics, Inc., Munich, Germany) using a Hitachi 7600-010 automatic analyzer (Hitachi, Tokyo, Japan). Plasma glucose levels were measured using the glucose oxidase technique (Roche Diagnostics, Mannheim, Germany). The intra- and inter-assay coefficients of variation for all measurements were less than 5% and 10%, respectively. The homeostatic model assessment of insulin resistance (HOMA-IR) was calculated as [fasting glucose concentration (mmol/L) × fasting insulin concentration (μU/mL)]/22.5. The atherogenic index (AI) was calculated using the following equation: AI = [(TC)-(HDL-C)]/[HDL-C] (39).

### Statistical Analysis

Data are presented as the mean ± standard deviation. Allele frequencies of *ABCA1* polymorphisms were estimated by gene counting. Hardy-Weinberg equilibrium was tested in cases and controls using a chi-square test. Allele and genotype frequencies were compared between cases and controls using chi-square analysis. Differences in variables between the GDM and control subjects were evaluated using independent-sample Student t-tests. To assess the effects of *ABCA1* gene polymorphism and changes in quantitative variables, we performed an analysis of variance. Adjustments for age and BMI were performed using analysis of covariance. The effects of other parameters, including the rs2230806 G/A genotypes of *ABCA1*, glycemic status (GDM = 1, control = 0), pre-pregnancy BMI, and BMI at delivery, on insulin, TG, TC, HDL-C, and LDL-C levels were evaluated by multivariate stepwise regression analyses. Statistical significance was set at *p* < 0.05. All statistical analyses were performed using the Statistical Program for Social Sciences (SPSS) 21.0 (IBM, Armonk, NY, USA).

## Results

### Clinical and Biochemical Characteristics

The comparison of clinical characteristics and biochemical parameters between the patients with GDM and controls is shown in **Table 1**. Compared to the controls, prepregnancy BMI, basic systolic blood pressure (SBP), and diastolic blood pressure (DBP) were found to be significantly higher, and weight gain during pregnancy and neonatal birth height were significantly lower in the patients with GDM. Women with GDM also had significantly higher fasting insulin and glucose concentrations, HOMA-IR scores, and TG levels, and lower LDL-C levels, AI values, and apo A1 levels compared with the control group after adjusting for differences in age and BMI at delivery. Of the 660 women with GDM, 71 women required insulin treatment and 589 women had dietary and exercise-based treatment only (**Table 1**). These results were generally consistent with the clinical and metabolic profiles frequently observed in GDM subjects, as observed in our recent study (40).

**Table 1:** Clinical and biochemical characteristics in patients with GDM and control women.

	GDM (n = 660)	Control (n = 1097)	P values
**Clinical characteristics**			
Age (years)	35.57 ± 4.03	34.54 ± 4.99	<0.001
Gestational age (weeks)	38.04 ± 3.04	38.47 ± 1.72	0.647
Parity	1.61 ± 0.53	1.62 ± 0.55	0.786
Prepregnancy BMI (kg/m^2)^	22.27 ± 3.11	21.23 ± 2.75	<0.001
Weight gain during pregnancy(kg)	11.45 ± 4.20	13.92 ± 4.25	<0.001
Delivery BMI (kg/m^2)^	26.83 ± 3.35	26.60 ± 2.78	0.102
SBP(mmHg)	115.83 ± 11.01	115.11 ± 9.99	0.124
DBP(mmHg)	72.76 ± 8.60	72.59 ± 7.75	0.649
Basic SBP	118.31 ± 10.65	111.22 ± 14.31	<0.001
Basic DBP	75.04 ± 9.81	70.52 ± 9.66	0.001
Neonatal birth height (cm)	49.60 ± 1.83	49.85 ± 1.926	0.006
Neonatal birth weight (g)	3335.11 ± 442.38	3369.13 ± 390.15	0.083
Insulin treatment (n)	71	0	
**Metabolic profile**			
Fasting Ins (pmol/L)	15.23 ± 21.06	13.28 ± 16.22	0.043
Fasting Glu (mmol/L)	4.38 ± 0.88	4.10 ± 0.83	<0.001
HOMA-IR	3.18 ± 5.24	2.02 ± 0.92	<0.001
Triglycerides(mmol/L)	3.89 ± 1.69	3.67 ± 1.47	0.004
TC(mmol/L)	5.98 ± 1.24	6.04 ± 1.09	0.236
HDL-C(mmol/L)	1.97 ± 0.45	1.97 ± 0.42	0.921
LDL-C(mmol/L)	2.98 ± 0.97	3.17 ± 0.98	<0.001
Atherogenic index (AI)	2.10 ± 0.57	2.16 ± 0.67	0.033
Apo A1 (g/L)	2.30 ± 0.41	2.36 ± 0.44	0.001
Apo B (g/L)	1.15 ± 0.26	1.15 ± 0.26	0.731

Values are present as mean ± SD.

### ABCA1 Genotype and Allele Frequencies

**Table 2** displayed the genotypic or allelic frequencies of SNP rs2230806 in both GDM patients and controls. All 1757 samples were successfully genotyped, and the frequencies of the SNP were in Hardy-Weinberg equilibrium in both the GDM patient and control groups. The G and A allele frequencies of the rs2230806 locus were 58.3% and 41.7%, respectively, in the GDM group and 55.9% and 44.1%, respectively, in the control group. These genotype and allele frequencies were not significantly different between groups (*p* > 0.05).

**Table 2:** Genotype and allele frequency for the rs2230806 SNP of the ABCA1 gene in women with GDM and control subjects.

	GDM (n = 660)	Control (n = 1097)	P
ABCA1			
Genotype			0.325
GG	33.2% (219)	29.8% (327)	
GA	50.1% (331)	52.2% (573)	
AA	16.7% (110)	18.0% (197)	
Allele			0.176
G	58.3% (769)	55.9% (1227)	
A	41.7% (551)	44.1% (967)	

numbers in parentheses indicate number of subjects with each genotype or number of alleles of each type.

In addition, when GDM patients and control subjects were further divided into overweight/obese and non-obese subgroups, no significant differences in genotype or allele frequencies were observed between these subgroups (**Table 3**).

**Table 3:** Genotype and allele frequency for the rs2230806 SNP of the ABCA1 gene in women with GDM and control subjects divided into overweight/obese and nonobese subgroups.

	GDM			Control		
	Overweight/Obese (n = 474)	Non-obese (n = 186)	P	Overweight/Obese (n = 805)	Non-obese (n = 292)	P
ABCA1						
Genotype			0.087			0.399
GG	34.8% (165)	29.0% (54)		28.9% (233)	32.2% (94)	
GA	47.5% (225)	57.0% (106)		52.3% (421)	52.1% (152)	
AA	17.7% (84)	14.0% (26)		18.8% (151)	15.8% (46)	
Allele			0.736			0.192
G	58.5 (555)	57.5% (214)		55.1% (887)	58.2% (340)	
A	41.5% (395)	42.5% (158)		44.9% (723)	41.8% (244)	

numbers in parentheses indicate number of subjects with each genotype or number of alleles of each type.

### Effect of ABCA1 SNP on Clinical and Metabolic Parameters of the GDM and Control Groups

Associations of the SNP rs2230806 and clinical characteristics and metabolic traits are shown in **Table 4**. In the GDM patient group, AA homozygotes at the rs2230806 locus had lower HDL-C levels and higher AI values than GG homozygotes and GA heterozygotes (*p* < 0.05). These differences remained significant after adjusting for age and BMI. There were no significant changes in other parameters according to the genotype at the rs2230806 locus in GDM patients.

**Table 4:** Clinical characteristics and metabolic profile of ABCA1 rs2230806 genotypes in GDM patients and controls.

	GDM			Control		
	GG (219)	GA (332)	AA (108)	GG (327)	GA (573)	AA (197)
**Clinical characteristics**					
Age (years)	36.05 ± 4.04	35.10 ± 3.95^*^ ^a^	35.47 ± 3.56	35.64 ± 3.82	35.95 ± 4.86	35.49 ± 3.44
Gestation age (weeks)	38.96 ± 1.00	39.00 ± 1.00	39.07 ± 0.83	39.16 ± 1.05	39.25 ± 0.94	39.24 ± 0.86
Prepregnancy BMI (kg/m^2^)	22.22 ± 2.74	22.29 ± 3.40	22.40 ± 3.00	21.04 ± 2.36	21.23 ± 2.71	21.26 ± 3.10
Weight gain during pregnancy (kg)	11.91 ± 4.38	11.17 ± 5.08^*^ ^a^	11.54 ± 4.10	13.89 ± 3.86	13.90 ± 4.53	14.07 ± 3.95
Delivery BMI (kg/m^2^)	26.88 ± 2.72	26.78 ± 3.80	26.91 ± 3.23	26.44 ± 2.56	26.68 ± 2.64	26.92 ± 2.97
Neonatal birth height (cm)	49.69 ± 1.77	49.61 ± 1.83	49.57 ± 1.65	49.85 ± 1.85	49.78 ± 1.87	49.90 ± 2.22
Neonatal birth Weight (g)	3345.37 ± 406.29	3348.17 ± 460.88	3322.23 ± 403.06	3365.12 ± 387.46	3373.44 ± 370.70	3368.89 ± 395.92
SBP (mmHg)	116.02 ± 12.34	115.70 ± 10.32	116.23 ± 10.81	115.86 ± 9.85	115.05 ± 10.14	114.89 ± 10.36
DBP (mmHg)	72.86 ± 8.55	72.42 ± 8.74	73.81 ± 8.33	71.95 ± 7.69	72.60 ± 7.73	71.63 ± 7.42
**Metabolic profile**						
Fasting Ins (μU/ml)	12.16 ± 13.19	14.35 ± 15.92	17.70 ± 28.86	10.46 ± 4.75	10.09 ± 4.41	10.72 ± 4.76
Fasting Glu (mmol/L)	4.76 ± 0.55	4.68 ± 0.63	4.85 ± 0.63	4.37 ± 0.35	4.38 ± 0.38	4.43 ± 0.34
HOMA-IR	2.78 ± 4.46	3.25 ± 5.10	4.24 ± 8.02	2.03 ± 0.93	1.97 ± 0.90	2.12 ± 0.95
Triglycerides (mmol/L)	3.79 ± 1.49	3.94 ± 1.69	3.90 ± 1.78	3.60 ± 1.24	3.74 ± 1.57	3.57 ± 1.35
TC (mmol/L)	5.93 ± 1.14	6.02 ± 1.07	5.97 ± 1.83	6.17 ± 1.08	6.11 ± 1.07	5.94 ± 1.11^*^ ^a^
HDL-C (mmol/L)	2.02 ± 0.45	2.00 ± 0.42	1.89 ± 0.48^*^ ^a^ ^.^ ^b^	2.03 ± 0.38	2.00 ± 0.42	1.97 ± 0.45
LDL-C (mmol/L)	2.98 ± 1.17	2.97 ± 0.83	2.87 ± 0.80	3.30 ± 1.11	3.18 ± 0.94	3.10 ± 0.90^*^ ^a^
Atherogenic index	2.00 ± 0.53	2.08 ± 0.55	2.20 ± 0.54^*^ ^a^ ^.^ ^b^	2.10 ± 0.60	2.13 ± 0.57	2.10 ± 0.62
apoA1 (g/L)	2.32 ± 0.40	2.28 ± 0.35	2.28 ± 0.40	2.39 ± 0.46	2.38 ± 0.42	2.33 ± 0.41
apoB (g/L)	1.14 ± 0.27	1.17 ± 0.25	1.13 ± 0.24	1.17 ± 0.27	1.15 ± 0.26	1.13 ± 0.27

a compared with GG genetype carriers in the same group.

b compared with GA genetype carriers in the same group *p<0.05.

In addition, in the control group, TC and LDL-C levels were lower in AA homozygotes than GG homozygotes (all *p* < 0.05, **Table 4**). There is an insignificant trend of genotype-related effect on HDL-C variation similar as that in GDM patients. There were no significant changes according to genotype for the other tested parameters in control subjects.

### Effect of ABCA1 SNP on Clinical and Metabolic Parameters in Overweight/Obese and Nonobese Subgroups

Since several polymorphisms of the ABCA1 gene affect HDL-C levels in the general population, and low HDL-C levels were associated with high BMI (obese phenotype), we further analyzed the effects of the *ABCA1* gene polymorphism on clinical and metabolic parameters in GDM patients and control subjects stratified into overweight/obese (BMI ≥ 25 kg/m^2^) and non-obese (BMI < 25 kg/m^2^) subgroups. In the overweight/obese GDM group, we found that AA homozygotes at the rs2230806 polymorphic site had higher DBP and AI values than heterozygotes and GG homozygotes, lower TC levels than heterozygotes, and lower HDL-C levels than heterozygotes and GG homozygotes (*p* < 0.05, **Table 5**). In addition, in the non-obese GDM group, the AA homozygotes had a lower pre-pregnancy BMI and delivery BMI, and higher TC and AI than GG homozygotes or GG homozygotes and GA heterozygotes (**Table 5**).

**Table 5:** Clinical characteristics and metabolic profile of ABCA1 rs2230806 genotypes in overweight/obese and non-obese GDM patients.

	Overweight/Obese GDM			Non-obese GDM		
	GG(165)	GA(225)	AA(84)	GG(54)	GA(106)	AA(26)
**Clinical characteristics**						
Age(years)	36.19 ± 3.91	35.58 ± 3.71	35.92 ± 3.45	35.63 ± 4.44	34.06 ± 4.27^*^ ^a^	34.00 ± 3.63
Gestation age(weeks)	38.96 ± 0.96	39.01 ± 0.91	39.13 ± 0.76	38.95 ± 1.07	38.97 ± 1.16	38.90 ± 1.03
Prepregnancy BMI (kg/m^2^)	23.05 ± 2.56	23.66 ± 3.15^*^ ^a^	23.47 ± 2.50	19.64 ± 1.37	19.35 ± 1.50	18.82 ± 1.25^*^ ^a^
Weight gain during pregnancy (kg)	12.48 ± 4.47	11.49 ± 4.23^*^ ^a^	11.97 ± 4.42	10.06 ± 3.58	10.47 ± 3.64	10.04 ± 2.39
Delivery BMI (kg/m^2^)	27.95 ± 2.22	28.34 ± 3.62	28.18 ± 2.49	23.62 ± 1.00	23.49 ± 1.18	22.81 ± 1.40^*^ ^a^ ^.^ ^b^
Neonatal birth height (cm)	49.75 ± 1.82	49.82 ± 1.80	49.60 ± 1.72	49.52 ± 1.62	49.18 ± 1.84	49.54 ± 1.45
Neonatal birth Weight (g)	3381.86 ± 404.55	3420.80 ± 462.54	3361.999 ± 407.56	3240.74 ± 397.81	3195.86 ± 420.96	3195.00 ± 376.05
SBP(mmHg)	116.45 ± 13.05	116.62 ± 10.30	117.64 ± 11.03	114.62 ± 9.91	113.70 ± 10.16	111.54 ± 8.94
DBP(mmHg)	72.92 ± 8.95	72.15 ± 8.59	75.11 ± 7.99^*^ ^b^	72.66 ± 7.31	72.93 ± 0.09	69.96 ± 8.32
**Metabolic profile**						
Fasting Ins (μU/ml)	12.95 ± 14.84	16.15 ± 18.18	20.75 ± 32.05	9.33 ± 5.84	10.70 ± 9.26	6.06 ± 2.34
Fasting Glu (mmol/L)	4.78 ± 0.59	4.70 ± 0.67	4.01 ± 0.65	4.69 ± 0.43	4.64 ± 0.55	4.61 ± 0.48
HOMA-IR	3.01 ± 5.11	3.71 ± 5.96	5.03 ± 8.94	1.98 ± 1.36	2.31 ± 2.42	1.25 ± 0.52
Triglycerides(mmol/L)	3.91 ± 1.56	4.08 ± 1.68	3.92 ± 1.75	3.44 ± 1.23	3.64 ± 1.69	3.84 ± 1.96
TC(mmol/L)	5.92 ± 1.19	5.97 ± 1.09	5.68 ± 1.07^*^ ^b^	5.96 ± 0.95	6.11 ± 1.04	6.91 ± 3.10^*^ ^a^ ^.^ ^b^
HDL-C(mmol/L)	1.99 ± 0.44	1.97 ± 0.41	1.81 ± 0.43^*^ ^a^ ^.^ ^b^	2.12 ± 0.48	2.05 ± 0.46	2.16 ± 0.58
LDL-C(mmol/L)	2.94 ± 0.98	2.90 ± 0.81	2.78 ± 0.81	3.12 ± 1.63	3.12 ± 0.87	3.15 ± 0.74
Atherogenic index	2.04 ± 0.55	2.08 ± 0.53	2.20 ± 0.49^*^ ^a^	1.89 ± 0.49	2.06 ± 0.61	2.19 ± 0.70^*^ ^a^
apoA1(g/L)	2.31 ± 0.41	2.29 ± 0.34	2.26 ± 0.40	2.35 ± 0.36	2.27 ± 0.37	2.36 ± 0.40
apoB(g/L)	1.15 ± 0.29	1.16 ± 0.25	1.11 ± 0.24	1.11 ± 0.22	1.19 ± 0.24	1.22 ± 0.23

a compared with GG genetype carriers in the same group.

b compared with GA genetype carriers in the same group *p < 0.05.

In the overweight/obese control group, TC, LDL-C, and apoB levels were lower in the AA homozygotes than the GG homozygotes (all *p* < 0.05). Genotype-related effects at this locus were not observed in non-obese control subjects (**Table 6**).

**Table 6:** Clinical characteristics and metabolic profile of ABCA1 rs2230806 genotypes in overweight/obese and non-obese control subjects.

	Overweight/Obese control			Non-obese control		
	GG (233)	GA (421)	AA (151)	GG (94)	GA (152)	AA (46)
**Clinical characteristics**						
Age (years)	35.83 ± 3.71	36.17 ± 5.19	35.62 ± 3.40	34.93 ± 4.06	35.34 ± 3.81	35.04 ± 3.64
Gestation age (weeks)	39.21 ± 0.99	39.27 ± 0.95	39.24 ± 0.84	39.04 ± 1.21	39.18 ± 0.88	39.19 ± 0.94
Prepregnancy BMI (kg/m^2^)	21.98 ± 2.15	22.11 ± 2.54	21.90 ± 3.18	18.70 ± 1.37	18.82 ± 1.34	19.03 ± 1.17
Weight gain during pregnancy (kg)	14.45 ± 3.99	14.43 ± 4.86	14.73 ± 3.95	12.32 ± 3.14	12.39 ± 2.97	11.81 ± 3.10
Delivery BMI (kg/m^2^)	27.65 ± 1.91	27.80 ± 2.09	27.95 ± 2.58	23.46 ± 1.16	23.57 ± 1.08	23.53 ± 0.95
Neonatal birth height (cm)	49.97 ± 1.87	49.90 ± 1.95	50.02 ± 2.18	49.54 ± 1.84	49.48 ± 1.62	49.53 ± 2.39
Neonatal birth Weight (g)	3397.63 ± 369.76	3409.30 ± 373.40	3407.53 ± 409.17	3278.28 ± 430.57	3272.76 ± 346.10	3249.58 ± 328.66
SBP (mmHg)	116.59 ± 9.90	115.72 ± 10.41	116.08 ± 9.97	114.26 ± 9.66	113.25 ± 9.17	111.39 ± 10.85
DBP (mmHg)	72.06 ± 7.57	72.93 ± 7.85	72.13 ± 7.32	71.63 ± 7.99	71.76 ± 7.36	70.00 ± 7.73
**Metabolic profile**						
Fasting Ins (μU/ml)	11.03 ± 4.99	10.56 ± 4.31	11.14 ± 4.99	8.71 ± 3.67	8.82 ± 4.46	9.14 ± 3.64
Fasting Glu (mmol/L)	4.39 ± 0.35	4.39 ± 0.38	4.42 ± 0.35	4.34 ± 0.32	4.33 ± 0.36	4.47 ± 0.32
HOMA-IR	2.15 ± 0.97	2.07 ± 0.88	2.21 ± 1.01	1.67 ± 0.69	1.70 ± 0.90	1.80 ± 0.70
Triglycerides (mmol/L)	3.64 ± 1.30	3.83 ± 1.64	3.65 ± 1.40	3.46 ± 1.08	3.47 ± 1.33	3.38 ± 1.31
TC (mmol/L)	6.14 ± 1.11	6.04 ± 1.06	5.85 ± 1.10^*^ ^a^	6.26 ± 1.03	6.31 ± 1.07	6.23 ± 1.12
HDL-C (mmol/L)	2.00 ± 0.37	1.96 ± 0.40	1.95 ± 0.44	2.11 ± 0.39	2.10 ± 0.44	2.03 ± 0.47
LDL-C (mmol/L)	3.31 ± 1.19	3.12 ± 0.93^*^ ^a^	3.01 ± 0.87^*^ ^a^	3.32 ± 0.91	3.36 ± 0.95	3.40 ± 0.96
Atherogenic index	2.13 ± 0.56	2.14 ± 0.55	2.09 ± 0.65	2.03 ± 0.65	2.09 ± 0.63	2.14 ± 0.51
apoA1 (g/L)	2.39 ± 0.48	2.37 ± 0.41	2.34 ± 0.43	2.40 ± 0.42	2.38 ± 0.45	2.29 ± 0.32
apoB (g/L)	1.17 ± 0.27	1.14 ± 0.25	1.11 ± 0.28^*^ ^a^	1.18 ± 0.27	1.19 ± 0.27	1.20 ± 0.25

a compared with GG genetype carriers in the same group *p < 0.05.

### Association of Important Independent Variables With Insulin and Lipid Levels in Women With and Without GDM

Based on multivariate stepwise regression analysis, **Table 7** showed that maternal BMI was a significant predictor of fasting insulin levels. In addition, prepregnancy BMI and glycemic status were significant predictors of TG and TC levels, prepregnancy BMI and allelic variant were significant predictors of HDL-C levels, and prepregnancy BMI, glycemic status, and allelic variant were significant predictors of LDL-C levels in women with and without GDM (**Table 7**).

**Table 7:** Association of important independent predictors/confounders with biomarker levels by multivariate regression analysis in women with and without GDM.

**Independents**	Unstandardized coefficients		Standardized coefficients	*t*	*p*
	*β*	S.E.M.	*β*		
Model I: Fasting insulin level as dependent variable					
Constant	-9.036	4.058		-2.227	0.026
Maternal BMI (kg/m^2^)	0.859	0.151	0.145	5.685	0.000
Model II: Triglyceride level as dependent variable					
Constant	2.879	0.334		8.610	0.000
prepregnancy BMI (kg/m^2^)	0.053	0.013	0.102	4.037	0.000
Glycemic status	-0.171	0.079	-0.055	-2.167	0.030
Model III: TC level as dependent variable					
Constant	4.131	0.268		15.442	0.000
prepregnancy BMI (kg/m^2^)	-0.037	0.010	-0.112	-3.708	0.000
Glycemic status	-0.158	0.063	-0.067	-2.517	0.012
Model IV: HDL-C level as dependent variable					
Constant	2.566	0.082		31.122	0.000
prepregnancy BMI (kg/m^2^)	-0.023	0.004	-0.161	-6.536	0.000
Allelic variant	-0.034	0.015	-0.054	-2.208	0.027
Model V: LDL-C level as dependent variable					
Constant	3.841	0.224		17.177	0.000
prepregnancy BMI (kg/m^2^)	-0.042	0.008	-0.123	-4.920	0.000
Glycemic status	0.208	0.051	0.102	4.091	0.000
Allelic variant	-0.086	0.035	-0.059	-2.414	0.016

Glycemic status (GDM+, GDM−).

## Discussion

In this study we used candidate gene approach to explore the association of a lipid-related gene rs2230806 SNP (R219K) with the risk of GDM and related traits. Our results showed, for the first time, that the polymorphism was significantly associated with HDL-C levels and AI values in GDM patients. Further subgroup analysis showed that the polymorphism was associated with DBP in addition to HDL-C levels and AI values in overweight/obese GDM patients, while it was associated with TC levels, AI values, prepregnancy BMI, and BMI at delivery in non-obese GDM patients. These results provide evidence that the polymorphism is associated with variations in atherometabolic traits in GDM patients, with characteristics of BMI dependency. These findings highlight a link between related phenotypes in women with GDM and the genetic component.

The rs2230806 polymorphism controls the substitution of an arginine at residue 219 of the peptide with a lysine. This substitution may affect the function of ABCA1; that is, the first step in reverse cholesterol transport (41). Our results in a Chinese cohort showed that the rs2230806 polymorphism was associated with HDL-C concentration in GDM patients, especially those with higher BMI, supporting the notion that ABCA1 plays a central role in reverse cholesterol transport as a modulator of HDL-C. In addition, the same trend of the genotype-related effect on HDL-C levels in the control group reflexes that the difference of the HDL-C level among genotypes was relatively small in this group of people, which is consisting with the result in a Japanese population reported by Harada et al. (42).

It is unclear why overweight/obesity modulated the effect of the polymorphism on HDL-C levels. A previous study showed that, compared with small adipocytes, large adipocytes contain reduced membrane cholesterol concentrations, and that cholesterol traffic genes, including *ABCA1*, are involved in the response to cholesterol depletion in the membranes (43). Another study reported that, compared with lean men, overweight men have a lower ability to promote ABCA1-mediated cholesterol efflux (44). In addition, insulin may reduce ABCA1 expression levels in adipocytes, resulting in insulin resistance (45). Therefore, ABCA1 levels are downregulated to a greater extent in overweight/obese GDM patients, who are usually hyperinsulinemic, than in normal-weight subjects. Thus, obesity or overweight may induce effects directly linked to ABCA1 function that may interact with the *ABCA1* polymorphism. However, the minor A allele was associated with decreased HDL-C levels in GDM patients, especially in those with overweight/obesity, which is in contrast to several previous reports in the general population (26, 27, 46). Similar results were also observed in obese non-pregnant subjects in the French D.E.S.I.R. study (47). The authors of the D.E.S.I.R. proposed that, when the minor A allele is associated with higher HDL levels, overweight/obesity cancels this effect, and when there is an additional minor allele associated with lower levels (as for the 378 C allele of rs1800978), overweight enhances this effect, leading to a lower HDL-C concentration outcome. We speculate that there might be a similar explanation for the association of the minor A allele with lower HDL-C levels in overweight/obese GDM patients in the present study.

While we observed an association between the *ABCA1* polymorphism and HDL-C levels in GDM patients, we also found a BMI-dependent relationship between this polymorphism and other lipid-related phenotypes, such as TC and/or LDL-C levels. In overweight/obese GDM patients, significantly lower TC levels were observed between patients with AA and GA genotypes (*p* < 0.05). In contrast, non-obese GDM subjects with the AA genotype showed increased TC levels compared to those with GG or GA genotypes (all *p* < 0.05). This difference may be attributed to the different BMI statuses of GDM patients. Several studies have shown that the A allele of this polymorphism is significantly associated with lower TC levels (29–31). The result observed in the overweight/obese GDM group in this study is in line with previous reports. The result observed in non-obese GDM women may be attributed to smaller BMI-mediated effect on subjects with the AA genotype, since in the non-obese GDM group, there were clear genotype-related effects on prepregnancy BMI and BMI at delivery. The relatively lower BMI in subjects with the AA genotype compared to those with the GG or GA genotypes may be linked to changes in ABCA1 levels (and thus, perhaps protein function). Indeed, a previous study demonstrated that the expression of ABCA1 is downregulated to a greater extent in obese subjects (relatively higher BMI) than in normal-weight subjects (relatively lower BMI), suggesting that BMI-related effects on cholesterol levels may also exist among non-obese GDM patients with different genotypes.

In addition, in control subjects with normal pregnancies, the AA genotype was associated with decreased TC and LDL-C levels compared with the GG genotype (both *p* < 0.05). This is consistent with the results of previous studies (28–33). When overweight/obese and non-obese subgroups were further divided, genotype-related effects were only evident in overweight/obese control subjects. This indicates that the genotype-related effect on these two cholesterol fractions may also be BMI-dependent in normal pregnant women.

In the current study, overweight/obese GDM patients with the AA genotype showed significantly higher DBP (75.11 ± 7.99 mmHg) than those with the GA genotype (72.15 ± 8.59 mmHg). However, this difference was not observed in non-obese subjects with GDM. Clinical diabetes, including GDM, is considered an independent risk factor for cardiovascular complications. Women with GDM have a higher risk of developing hypertensive disorders or preeclampsia. One study using transcriptomics showed in the placenta a high level of pro-inflammatory factor expression with signs of major vascular dysfunction (48). Another study found increased levels of an array of pro-inflammatory molecules (49, 50), as well as a pro-inflammatory pattern of upregulated adipokines and decreased levels of the anti-inflammatory factor, adiponectin, in adipose tissue from women with GDM (51). This pro-inflammatory milieu, together with the dysregulated secretion of placental factors and/or β-cell injury, may trigger metabolic and cardiovascular abnormalities, including hypertension, in women with GDM and their offspring (51).

Several studies have established an association between lipid-related gene polymorphisms and blood pressure variation or hypertension in different populations and ethnic groups. For example, one study reported that the genotype frequencies of *ABCA1* V825I were different between normotensive and hypertensive women, and DBP differed according to *ABCA1* genotype (52). Yin et al. also reported that this polymorphism, as well as several other lipid-related gene polymorphisms, interact with overweight/obesity to modulate blood pressure (SBP, DBP and pulse pressure) levels (53). In addition, another report demonstrated that the -14 C→T polymorphism of the *ABCA1* gene is a determinant of blood pressure and the development of hypertension in Japanese individuals (54), and nine lipid-related SNPs have interactions with the effect of cigarette smoking on blood pressure levels (55). These findings, together with our current study results, indicate the importance of lipid-related gene polymorphisms (including *ABCA1* SNPs) in mediating blood pressure variation, and modifying the additional interaction of components such as overweight/obese and smoking.

In the present study, we also found an association between the *ABCA1* rs2230806 SNP and AI value in all GDM patients and in individual overweight/obese and non-obese subgroups. AI values were higher in AA homozygotes in the GDM group and its separate subgroups than in GG homozygotes (**Tables 4** and **5**, all *p* < 0.05). These data strongly support the notion that patients with GDM have a higher risk of CAD and suggest that the genetic component is associated with atherogenic risk markers, including the AI values.

Several epidemiologic studies have shown positive associations between maternal gestational weight gain (GWG) and prepregnancy BMI and cardiometabolic risk factors in the offspring, such as fat mass (56), weight trajectory (57), waist circumference, BMI, and blood pressure (58). Recent epigenetic epidemiological studies suggest that low maternal prepregnancy BMI (59, 60) and GWG (61) are related to differential peripheral blood DNA methylation in offspring, indicating the importance of maternal prepregnancy BMI on the potential life-long health of offspring. In this study, we found an association between the *ABCA1* rs2230806 SNP and prepregnancy BMI variation, which suggests that the maternal genetic component may have implications in determining the cardiometabolic health of the offspring by affecting maternal prepregnancy BMI.

Additionally, we have analyzed our data by regression analysis. The results demonstrated that prepregnancy BMI was an important predictor of TG, TC, HDL-C, and LDL-C levels, while the allelic variant, and both the allelic variant and glycemic status were also important predictors of HDL-C and LDL-C levels, respectively (**Table 7**). These results highlight the importance of prepregnancy BMI in determining these lipid phenotypes, and the genetic component in modulating HDL-C and LDL-C concentrations. Moreover, these results suggest that *ABCA1* genetic variation was significantly associated with some lipid abnormalities in women with GDM and with metabolic adaption in normal pregnancies.

Our study has some strengths and limitations that should be noted. The strengths of this study are as follows. First, the study included both maternal and fetal/neonatal data. Second, the study adds to the limited body of evidence regarding the genetics of hyperglycemia during pregnancy. Third, we provided insights into the genetic regulator of lipid metabolism and its effect on biomarkers of insulin resistance in human pregnancy. The study limitations include a lack of data on *ABCA1* expression levels according to different *ABCA1* genotypes, which would have provided further insights into the mechanisms responsible for the genetic association. In addition, the sample size included in this study is relatively small. Moreover, our findings were limited to descriptive characteristics.

In conclusion, our study found no evidence that the *ABCA1* rs2230806 SNP is associated with GDM in Southwest Chinese women. However, it demonstrated that the rs2230806 polymorphism in the *ABCA1* gene is associated with HDL-C levels and AI values in GDM patients, and with TC and LDL-C levels in control subjects. In addition, a subgroup analysis showed that this SNP was also associated with DBP levels in addition to HDL-C levels and AI values in overweight/obese GDM patients, while it was associated with TC levels, AI values, pre-pregnancy BMI, and BMI at delivery in non-obese GDM women. Moreover, this SNP was associated with TC, LDL-C, and apoB levels in overweight/obese control subjects. Our results suggest that the rs2230806 polymorphism may be associated with increased maternal cardiovascular risk in women with GDM, and thus potentially, with an increased future risk of type 2 diabetes and CHD in mothers and their offspring. Screening for this variant in GDM patients, especially those with different BMI levels, and weight loss through changes in diet and lifestyle in women with GDM, may be beneficial and have preventive effects against complications in these patients.

## Data Availability Statement

The datasets for this study can be found at the found here:ABCA1 (rs2230806): https://www.ncbi.nlm.nih.gov/snp/rs2230806.

## Ethics Statement

The studies involving human participants were reviewed and approved by The Institutional Review Board of West China Second University Hospital, Sichuan University. The patients/participants provided their written informed consent to participate in this study.

## Author Contributions

HB conceived and designed the experiments, analyzed the data and revised the paper. FT performed experiments and wrote the paper. LG and XL was responsible for patient screening. MZ, YW, RL, YL, and SL helped with the experiments. PF and DL helped with the experiments and revised the paper. All authors have read and approved the final manuscript.

## Funding

This study was supported by grants from the National Natural Science Foundation of China 39870749, the National Key Research and Development Program of China 2016YFC1000400, Program for Changjiang Scholars and Innovative Research Team in University IRT0935, the Fundamental Research Funds for the Central Universities, and Research Seed Fund from West China Second University Hospital of Sichuan University (HB).

## Conflict of Interest

The authors declare that the research was conducted in the absence of any commercial or financial relationships that could be construed as a potential conflict of interest.

## Publisher’s Note

All claims expressed in this article are solely those of the authors and do not necessarily represent those of their affiliated organizations, or those of the publisher, the editors and the reviewers. Any product that may be evaluated in this article, or claim that may be made by its manufacturer, is not guaranteed or endorsed by the publisher.
